# Drought tolerance of sugarcane propagules is improved when origin material faces water deficit

**DOI:** 10.1371/journal.pone.0206716

**Published:** 2018-12-26

**Authors:** Fernanda C. C. Marcos, Neidiquele M. Silveira, Paulo E. R. Marchiori, Eduardo C. Machado, Gustavo M. Souza, Marcos G. A. Landell, Rafael V. Ribeiro

**Affiliations:** 1 Laboratory of Crop Physiology, Department of Plant Biology, Institute of Biology, University of Campinas (UNICAMP), Campinas, SP, Brazil; 2 Laboratory of Plant Physiology ‘Coaracy M. Franco’, Centre for Research and Development in Ecophysiology and Biophysics, Agronomic Institute (IAC), Campinas, SP, Brazil; 3 Department of Biology, Federal University of Lavras (UFLA), Lavras, MG, Brazil; 4 Department of Botany, Institute of Biology, Federal University of Pelotas (UFPel), Pelotas, RS, Brazil; 5 Sugarcane Research Center, IAC, Ribeirão Preto, SP, Brazil; University of the West Indies, TRINIDAD AND TOBAGO

## Abstract

Drought stress can imprint marks in plants after a previous exposure, leading to plant acclimation and a permissive state that facilitates a more effective response to subsequent stress events. Such stress imprints would benefit plants obtained through vegetative propagation (propagules). Herein, our hypothesis was that the propagules obtained from plants previously exposed to water deficit would perform better under water deficit as compared to those obtained from plants that did not face stressful conditions. Sugarcane plants were grown under well-hydrated conditions or subjected to three cycles of water deficit by water withholding. Then, the propagules were subjected to water deficit. Leaf gas exchange was reduced under water deficit and the propagules from plants that experienced water deficit presented a faster recovery of CO_2_ assimilation and higher instantaneous carboxylation efficiency after rehydration as compared to the propagules from plants that never faced water deficit. The propagules from plants that faced water deficit also showed the highest leaf proline concentration under water deficit as well as higher leaf H_2_O_2_ concentration and leaf ascorbate peroxidase activity regardless of water regime. Under well-watered conditions, the propagules from plants that faced stressful conditions presented higher root H_2_O_2_ concentration and higher activity of catalase in roots as compared to the ones from plants that did not experience water shortage. Such physiological changes were associated with improvements in leaf area and shoot and root dry matter accumulation in propagules obtained from stressed plants. Our results suggest that root H_2_O_2_ concentration is a chemical signal associated with improved sugarcane performance under water deficit. Taken together, our findings bring a new perspective to the sugarcane production systems, in which plant acclimation can be explored for improving drought tolerance in rainfed areas.

## Introduction

As a semi-perennial species, sugarcane plants face seasonal drought under field conditions, where water deficit causes reduction in photosynthesis and accumulation of carbohydrates, changes in antioxidant metabolism, and finally impairment of plant growth and sucrose yield [1, 2]. However, recurrent cycles of drought followed by rehydration are known to improve plant performance during a new stressful event [3–5]. Such phenomenon indicates that plants are able to change their metabolism and growth after an external stimulus, improving recovery or resilience of photosynthesis, increasing water use efficiency [4] and photoprotection [6] and reducing the negative impact of drought on yield, i.e. increasing resistance [7].

From a physiological perspective, plant acclimation is assumed as a functional change induced by an exogenous factor, such as water deficit [8]. Acclimation may result in improved plant response to stressors and then be related to stress memory, a way to store information of stressful events [3, 9, 10]. The physiological acclimation permits plants to overcome resource limitation, improving tolerance while impairing plant growth [8]. Such plant acclimation can imprint signals that remain after perturbation and affect the future plant response to biotic or abiotic stresses. While increased activity of antioxidant enzymes would permit a faster and more effective control of stress-induced production of reactive oxygen species [11], lower stomatal conductance due to accumulation of abscisic acid could reduce water consumption and improve water balance under drought without impacting photosynthetic rates [5].

Plants can sense the changing environment with all their body and their intricate cell signaling system, perceiving one stimulus in one site with the respective response being found in a different organ [12]. One important requirement for retaining information about previous limiting conditions is that the stress-induced signals are still present when the stressor is no longer affecting plants [13]. There is reasonable evidence for assuming that plants can sense changes in the environment during growth and modify the phenotype of their progenies to be more adapted to growth conditions [8, 14]. The stress-induced signals can be transferred to subsequent generations by seeds and remain active through vegetative propagation [15]. Accordingly, the clonal plants produced by vegetative propagation have apparently better ability to recover those stress-induced signals and improve their performance under limiting conditions as compared to the non-clonal plants [16].

Considering the drought-induced effects on plants propagated vegetatively, we hypothesized that the plants obtained from others previously exposed to drought will perform better under water deficit as compared to the propagules obtained from plants that never faced water shortage. Through vegetative propagation, information about previous stresses can be stored in sugarcane buds, which will sprout and produce new plants. Sugarcane is an important crop for ethanol and bioenergy production–a clean alternative for energy production–and its expansion to rainfed areas needs more drought tolerant plants. Then, exploring plant acclimation and signaling mechanisms would be an interesting tool for improving crop establishment and initial growth in such new areas.

## Materials and methods

### Plant material and growth conditions

Sugarcane (*Saccharum* spp.) plants cv. IACSP94-2094 were obtained from mini-stalks containing one bud and grown in plastic pots (0.5 L), with commercial substrate composed of sphagnum peat, expanded vermiculite, limestone dolomite, agricultural gypsum and NPK fertilizer (Carolina Soil, Vera Cruz RS, Brazil). Thirty-four days after planting, the plants were transferred to larger pots (20 L) containing typical red-yellow Latosoil [17], fertilized with urea (equivalent to 300 kg N ha^-1^), superphosphate (equivalent to 300 kg P_2_O_5_ ha^-1^) and potassium chloride (equivalent to 260 kg K_2_O ha^-1^) according to Dias and Rossetto [18]. During the experiment, other three fertilizations were performed at 30, 60 and 150 days after planting, with the same amount of urea, superphosphate and potassium chloride as the first fertilization. The plants were grown under greenhouse conditions, where the average air temperature was 24.4±6.6°C, relative humidity was 76±17% and the maximum photosynthetic photon flux density (PPFD) was approximately 1,200 μmol m^–2^ s^–1^. The plants were irrigated daily and grown under well-hydrated conditions until they were six-month old.

### Inducing water deficit to the origin material

When the plants were 6-month old, one group of plants was maintained under daily irrigation (W) and another group was subjected to three cycles of water deficit (D) by water withholding. Each cycle of water deficit lasted nine days and soil moisture was monitored with soil moisture-sensors model Water Scout SM100 (Yara ZimTechnology, Berlin, Germany). While the soil volumetric water content (VWC) reached 20% during the cycles of water deficit, it was higher than 60% in well-watered pots. After nine days of water deficit, the plants were irrigated and maintained under well-watered conditions for six days before the new cycle of water deficit, with leaf gas exchange being measured daily. After the three cycles of water deficit, we evaluated the number of tillers, the number of green and senescent leaves, the total leaf area and the dry matter of leaves, stems and roots. Then, the new plants–here named as propagules–were produced through vegetative propagation from those plants that experienced or not cycles of water deficit, as cited in the previous section.

### Inducing water deficit to propagules

After sprouting in commercial substrate (Carolina Soil, Vera Cruz RS, Brazil), the one-month old plants were placed in plastic boxes (12 L) with nutrient solution and transferred to a growth chamber (PGR15, Conviron, Winnipeg MB, Canada) under air temperature of 30/20°C (day/night), with 12 h photoperiod, air relative humidity of 80% and PPFD of 800 μmol m^-2^ s^-1^. Only the root system was immersed in modified Sarruge [19] nutrient solution (15 mmol L^-1^ N [7% as NH_4_^+^]; 4.8 mmol L^-1^ K; 5.0 mmol L^-1^ Ca; 2.0 mmol L^-1^ Mg; 1.0 mmol L^-1^ P; 1.2 mmol L^-1^ S; 28.0 μmol L^-1^ B; 54.0 μmol L^-1^ Fe; 5.5 μmol L^-1^ Mn; 2.1 μmol L^-1^ Zn; 1.1 μmol L^-1^ Cu and 0.01 μmol L^-1^ Mo). The nutrient solution was renewed weekly and its pH and electrical conductivity were maintained at 5.8±0.2 and 1.72±0.18 mS cm^-1^, respectively. The osmotic potential of the nutrient solution was -0.12 MPa. Two boxes containing the propagules obtained from irrigated plants and two boxes containing the propagules from plants subjected to three cycles of water deficit were prepared.

Forty-eight days after transferring plants to the hydroponic system, one group of plants was subjected to water deficit by adding PEG-8000 (Carbowax PEG-8000, Dow Chemical Comp, Midland MI, USA) to the nutrient solution for nine days. We added the PEG-8000 gradually to prevent osmotic shock. Then, the osmotic potential of the nutrient solution was reduced to -0.27, -0.57 and -0.77 MPa in three consecutive days. After nine days, the plants were recovered by supplying them with a nutrient solution with osmotic potential of -0.12 MPa (control condition) for five days. At the end, four treatments were defined taking into account the origin material and also the water regime that the propagules were facing: the propagules obtained from plants grown under well-watered conditions and then maintained under well-watered conditions (W/W); the propagules obtained from plants grown under well-watered conditions and then subjected to water (W/D); the propagules obtained from plants that experienced water deficit and then maintained under well-watered conditions (D/W); the propagules obtained from plants that faced water deficit and then subjected to water (D/D).

### Leaf gas exchange and photochemistry

Leaf gas exchange was measured daily with an infrared gas analyzer (LI-6400, LICOR, Lincoln NE, USA) attached to a modulated fluorometer (6400–40 LCF, LICOR, Lincoln NE, USA). The measurements were performed between 10:00 and 13:00 h under PPFD of 2,000 μmol m^−2^ s^−1^ and air CO_2_ concentration of 380 μmol mol^−1^. CO_2_ assimilation (*A*), stomatal conductance (*g*_S_), intercellular CO_2_ concentration (*C*_i_), transpiration (*E*), intrinsic water use efficiency (*A*/*g*_S_), and the instantaneous carboxylation efficiency (*k* = *A*/*C*_i_) were evaluated in fully expanded leaves. *A* and *E* values were integrated throughout the experimental period to estimate the total CO_2_ gain (*A*_i_), the total H_2_O loss through transpiration (*E*_i_), and the integrated water use efficiency (WUE = *A*_i_/*E*_i_). The integrated values were estimated assuming that the values measured between 10:00 and 13:00 h were constant during the 12 hours of photoperiod. The chlorophyll fluorescence was measured simultaneously to the leaf gas exchange and the apparent electron transport rate (ETR) was estimated as ETR = ɸ_PSII_× PPFD × 0.85 × 0.4, in which ɸ_PSII_ is the effective quantum efficiency of photosystem II (PSII), 0.85 is the light absorption and 0.4 is the fraction of light energy partitioned to PSII [20, 21]. Additionally, the non-photochemical quenching of fluorescence (NPQ) was evaluated and ETR/*A* calculated. In leaf tissues adapted to darkness (30 min), the potential quantum efficiency of photosystem II (*F*_V_/*F*_M_) was estimated [20].

### Leaf water potential and relative water content

The leaf water potential (ψ) was evaluated at the predawn with a pressure chamber (model 3005, Soilmoisture Equipment Corp., Santa Barbara CA, USA). The leaf relative water content RWC) was calculated using the fresh (FW), turgid (TW) and dry (DW) weights of leaf discs [22]: RWC = 100×(FW−DW)/(TW−DW). Both variables were measured at the maximum stress condition (9^th^ day of water deficit) and at the recovery period.

### Carbohydrates and proline

The extraction of total soluble carbohydrates (SS) was done with the methanol:chloroform:water solution [23] and quantified by the phenol–sulfuric acid method [24]. The sucrose content was quantified following van Handel [25] and the starch content (Sta) was determined by the enzymatic method proposed by Amaral et al. [26]. The concentration of nonstructural carbohydrates (NSC) in leaves and roots was calculated as NSC = SS+Sta. The total NSC was calculated considering the dry matter of each plant (mg plant^-1^). The plant nonstructural carbohydrates were calculated by the sum of leaf and root carbohydrates and carbohydrate partitioning among sugar types was also evaluated in both organs.

The leaf proline content was determined in test tubes by the reaction with the ninhydrin reagent (ninhydrin, acetic acid and orthophosphoric acid), glycine and acetic acid for 35 minutes at 100°C. The reaction mixture was extracted with toluene and the proline concentration was determined from a standard curve [27].

### Hydrogen peroxide and antioxidant enzymes

The evaluation of hydrogen peroxide (H_2_O_2_) was performed in 0.16 g fresh tissue (leaves and roots) ground in liquid nitrogen with the addition of polyvinylpolypyrrolidone (PVPP) and 0.1% of trichloroacetic acid (TCA) solution (w/v) [28]. The extract was centrifuged at 12,000 *g*, 4°C for 15 min. The crude extract was added to the reaction medium (1.2 mL of KI 1 mol L^−1^, potassium phosphate buffer pH 7.5 and 0.1 mol L^−1^) in microtubes and incubated on ice under dark for 1 h. After this period, the absorbance was evaluated at 390 nm. The calibration curve was done with H_2_O_2_ and results were expressed as μmol g^−1^ FW.

Enzymes were extracted from 0.2 g of fresh tissues of leaves and roots grounded in liquid nitrogen, with 1% of PVPP and 2 mL of extraction medium composed by 0.1 mol L^−1^ potassium phosphate buffer (pH 6.8), 0.1 mmol L^−1^ ethylenediaminetetraacetic (EDTA) and 1 mmol L^−1^ phenylmethylsulfonyl fluoride (PMSF). This homogenate was centrifuged at 15,000 *g* for 15 min and 4°C and the supernatant was collected and preserved on ice.

The superoxide dismutase (SOD, EC 1.15.1.1) activity was evaluated in a reaction medium with 3 mL of 100 mmol L^−1^ sodium phosphate buffer (pH 7.8), 50 mmol L^−1^ methionine, 5 mmol L^−1^ EDTA, deionized water, crude extract, 100 μmol L^−1^ riboflavin and 1 mmol L^−1^ nitro blue tetrazolium chloride (NBT). A group of tubes was exposed to light (fluorescent lamp of 30 W) for 15 min, and another group remained in darkness. The absorbance was measured at 560 nm and one unit of SOD is the amount of enzyme required to inhibit the NBT photoreduction in 50% [29]. SOD was expressed as U g^-1^ FW min^−1^.

The catalase (CAT, EC 1.11.1.6) activity was assayed in a reaction medium of 3 mL of 100 mmol L^−1^ potassium phosphate buffer (pH 6.8), deionized water, 125 mmol L^−1^ H_2_O_2_ and crude extract. The decrease in absorbance at 240 nm was measured and CAT activity was estimated using a molar extinction coefficient of 36 M^−1^ cm^−1^ and expressed as nmol g^−1^ FW min^−1^ [30].

For the ascorbate peroxidase (APX, EC 1.11.1.11) activity, the reaction medium was composed by 3 mL of 100 mmol L^−1^ potassium phosphate buffer (pH 6.0), deionized water, 10 mmol L^−1^ ascorbic acid, 10 mmol L^−1^ H_2_O_2_ and crude extract. The decrease in absorbance at 290 nm was measure and we used a molar extinction coefficient of 2.8 M^−1^ cm^−1^ to estimate APX in nmol g^−1^ FW min^−1^ [31].

### Biometry

The total leaf area was measured using the LI-3000 leaf area meter (LICOR, Lincoln NE, USA), and the shoot and root dry matter were evaluated after drying samples in a forced air oven at 65°C. Measurements were taken at the end of the experimental period.

### Statistical analysis

The experimental design was in randomized blocks, with four blocks and one plant per treatment in each block. The causes of variation were water conditions (two levels) and material origin (two levels) and the data were subjected to two-way ANOVA procedure. Mean values (n = 4 for each treatment) were compared by the Tukey test at 5% probability level.

## Results

### Origin plants under water deficit

Herein, the origin plants are defined as those ones that provided vegetative material for propagation, i.e., small stalk segments with buds. The origin plants were subjected to three cycles of water deficit and leaf gas exchange was measured during the dehydration and rehydration stages (S1 Fig). There was a significant reduction in leaf CO_2_ assimilation after four days of water withholding in all cycles of water deficit (S1 Fig), with the net photosynthesis reaching null values or even negative ones (respiration). Full recovery of leaf CO_2_ assimilation was noticed in all cycles and the negative impact of water deficit was reduced from the first to the third cycle (S1 Fig). After three cycles of water deficit, there was a significant reduction of biomass production (S2 Fig), with decreases in the number, dry matter and area of green leaves as well as decreases in the root and stem dry matter (S1 Table).

Then, small stalk segments (around 3 cm) with one bud were obtained from those plants and planted in individual recipients to produce new plants, i.e., the propagules. Buds from the plants subjected to water deficit had higher sprouting (*ca*. 95%) than buds from the plants maintained under well-watered conditions (*ca*. 74%). Thirty days after planting, the propagules were placed in plastic boxes with nutrient solution and four treatments were done after 18 days: the propagules from plants grown under well-watered conditions maintained under well-watered conditions (W/W) or subjected to water deficit (W/D); and the propagules from plants grown under cycles of water deficit maintained under well-watered conditions (D/W) or subjected to water deficit (D/D).

### Propagules under water deficit

**Fig 1:**
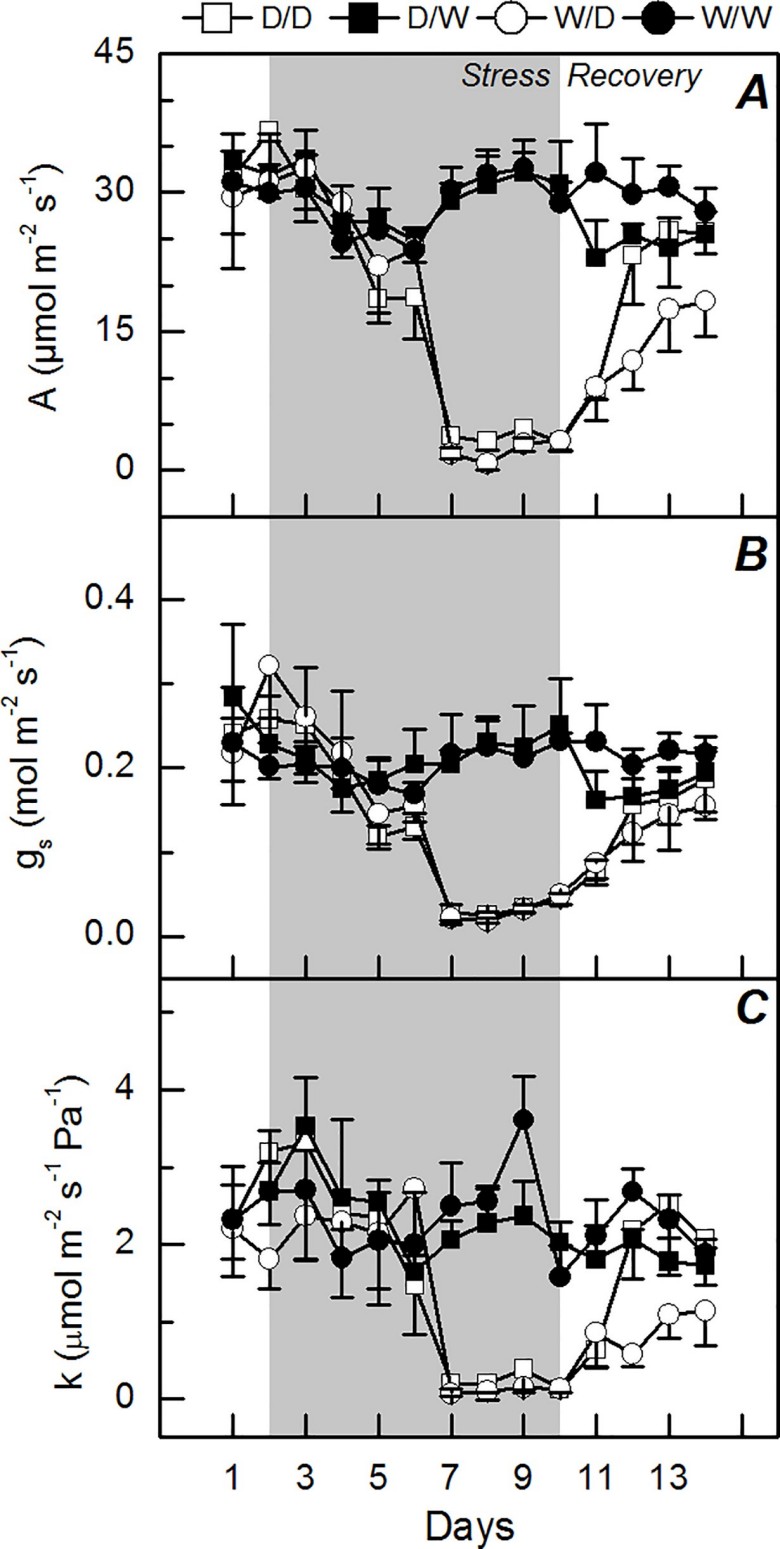
Time course of leaf gas exchange in sugarcane propagules under water deficit. Leaf CO_2_ assimilation (*A*), stomatal conductance (*B*), and instantaneous carboxylation efficiency (*C*) in sugarcane plants grown under well-watered conditions (W/W and D/W) or subjected to water deficit (W/D and D/D). The propagules were obtained from plants previously exposed to water deficit (D/W and D/D) or grown under well-watered conditions (W/W and W/D). The gray area indicates the water deficit period. Each symbol is the mean value ± s.d. (n = 4).

**Fig 2:**
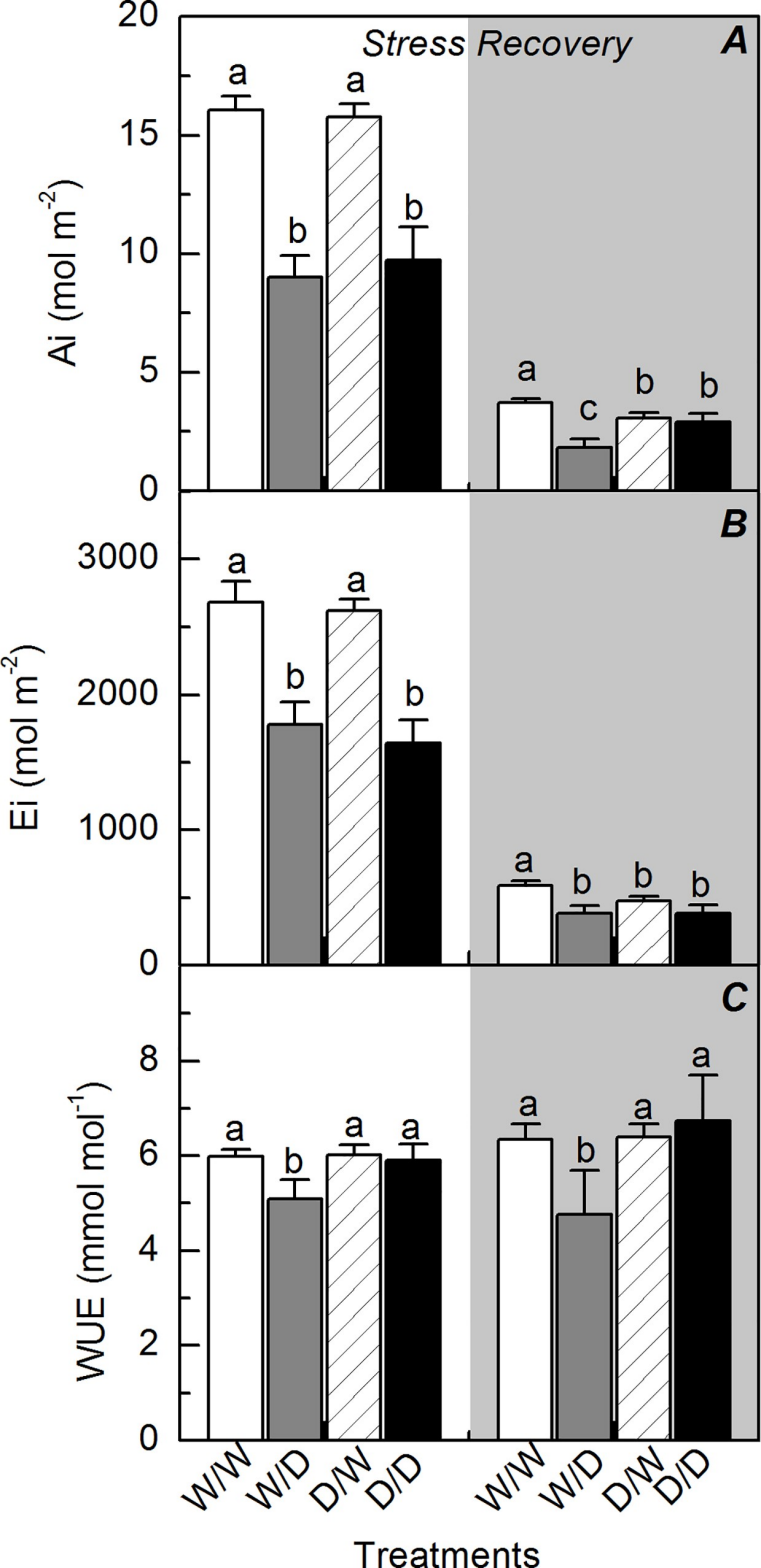
Integrated leaf gas exchange in sugarcane propagules during and after water deficit. The integrated CO_2_ assimilation (*A*), transpiration (*B*) and water use efficiency (*C*) in sugarcane plants grown under well-watered conditions (W/W and D/W) or subjected to water deficit (W/D and D/D). The propagules were obtained from plants previously exposed to water deficit (D/W and D/D) or grown under well-watered conditions (W/W and W/D). The integration was done during the water deficit (stress) and recovery (gray area) periods, as shown in Fig 1. Each histogram is the mean value + s.d. (n = 4). Different letters mean statistical differences among treatments (p<0.05).

Water deficit reduced the leaf CO_2_ assimilation, stomatal conductance and instantaneous carboxylation efficiency, regardless of the plant origin (Fig 1). Interestingly, the propagules originated from plants that experienced water deficit (D/D) presented a faster recovery of leaf CO_2_ assimilation and carboxylation efficiency as compared to W/D plants (Fig 1A and 1C). The integrated leaf CO_2_ assimilation and transpiration were reduced by water deficit in a similar way when comparing W/D and D/D treatments (Fig 2A and 2B). However, the recovery of photosynthesis was favored in D/D plants and then the integrated water use efficiency was improved under water deficit (Fig 2C).

**Fig 3:**
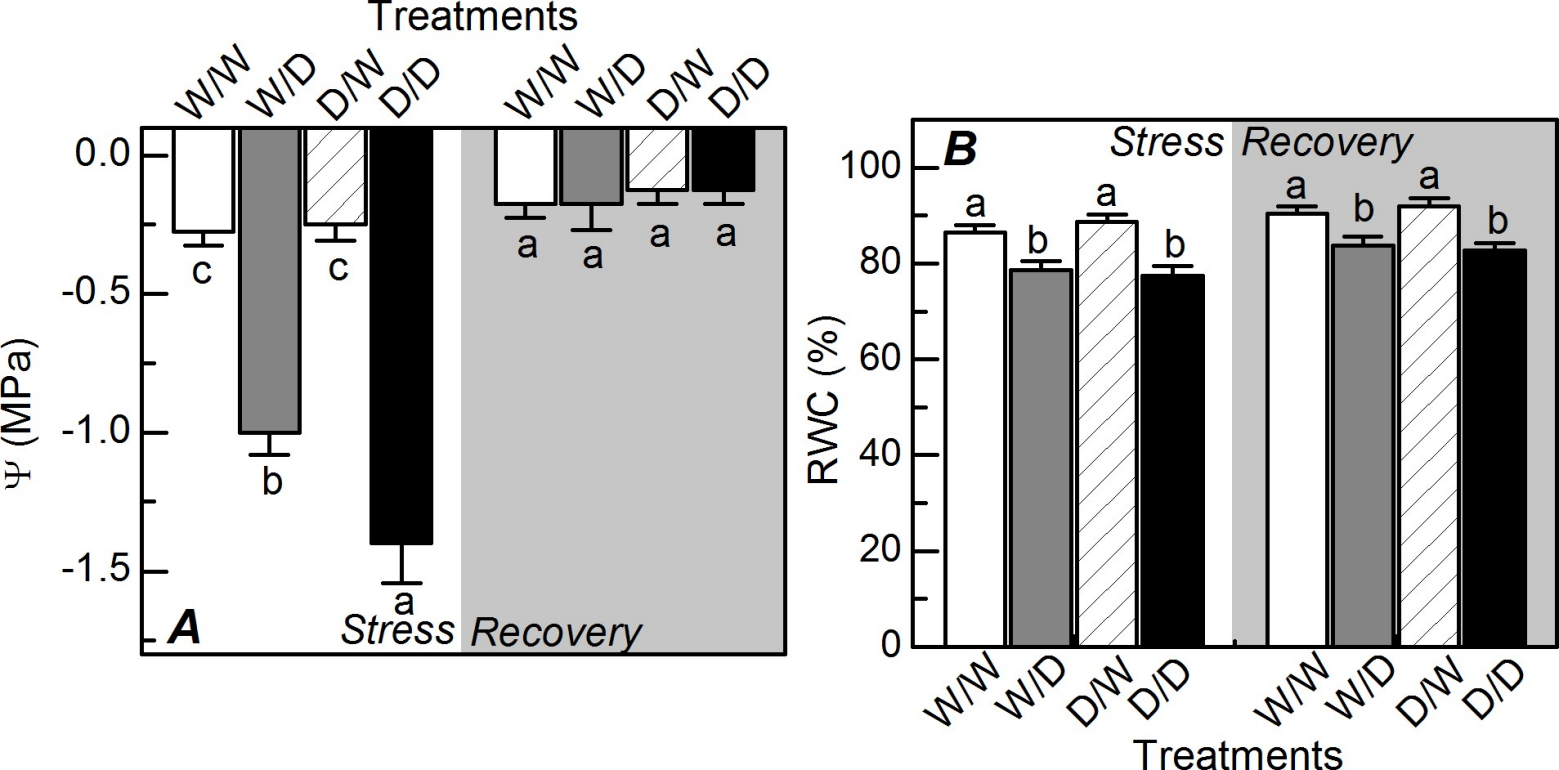
Water relations in sugarcane propagules under water deficit. The pre-dawn leaf water potential (*A*) and relative water content (*B*) in sugarcane plants grown under well-watered conditions (W/W and D/W) or subjected to water deficit (W/D and D/D). The propagules were obtained from plants previously exposed to water deficit (D/W and D/D) or grown under well-watered conditions (W/W and W/D). Measurements were done during the water deficit (stress) and recovery (gray area) periods, as shown in Fig 1. Each histogram is the mean value + s.d. (n = 4). Different letters mean statistical differences among treatments (p<0.05).

After nine days of water deficit, the pre-dawn leaf water potential was reduced and D/D plants showed the lowest values (Fig 3A). Regarding the leaf relative water content, there was a similar response to water deficit and both W/D and D/D plants exhibited the lowest values (Fig 3B). While the pre-dawn leaf water potential was fully recovered, the leaf relative water content was partially recovered after four days of plant rehydration (Fig 3).

**Fig 4:**
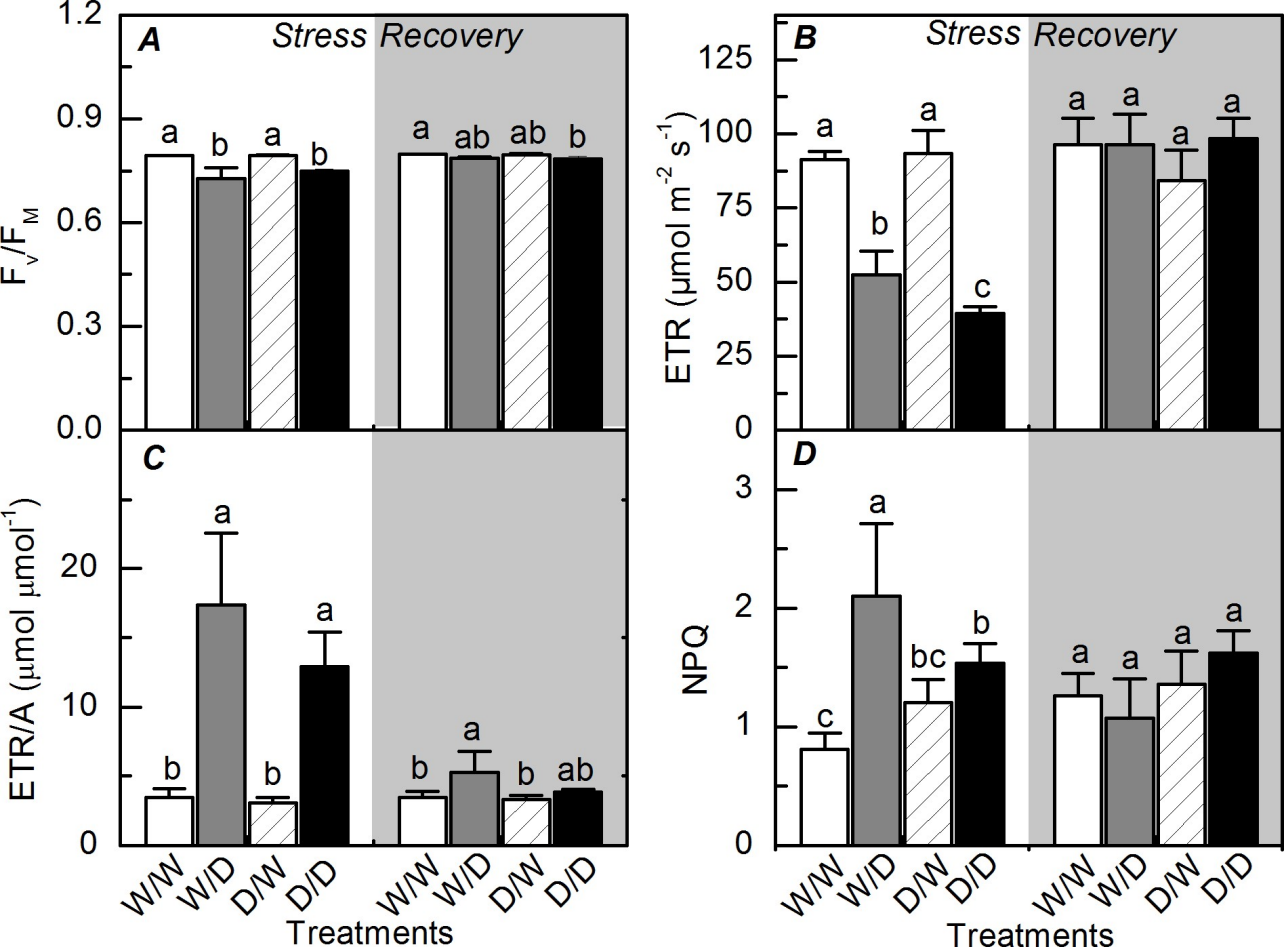
Photochemistry of sugarcane propagules during and after water deficit. The potential quantum efficiency of photosystem II (*A*), the apparent electron transport rate estimated (*B*), ETR/A ratio (*C*), and the non-photochemical quenching of fluorescence (D) in sugarcane plants grown under well-watered conditions (W/W and D/W) or subjected to water deficit (W/D and D/D). The propagules were obtained from plants previously exposed to water deficit (D/W and D/D) or grown under well-watered conditions (W/W and W/D). Measurements were done during the water deficit (stress) and recovery (gray area) periods, as shown in Fig 1. Each histogram is the mean value + s.d. (n = 4). Different letters mean statistical differences among treatments (p<0.05).

Water deficit caused decreases in the potential quantum efficiency of PSII (*F*_v_/*F*_m_) and also in the apparent electron transport rate (ETR) of W/D and D/D plants (Fig 4A and 4B). Although D/D plants had shown the lowest ETR values, the ratio ETR/*A* was similar between W/D and D/D plants, increasing in more than three times due to water deficit (Fig 4C). The non-photochemical quenching was increased by water deficit only in W/D plants (Fig 4D). All photochemical indices were recovered after plant rehydration, with W/W *vs*. W/D and D/W *vs*. D/D plants showing similar values.

**Fig 5:**
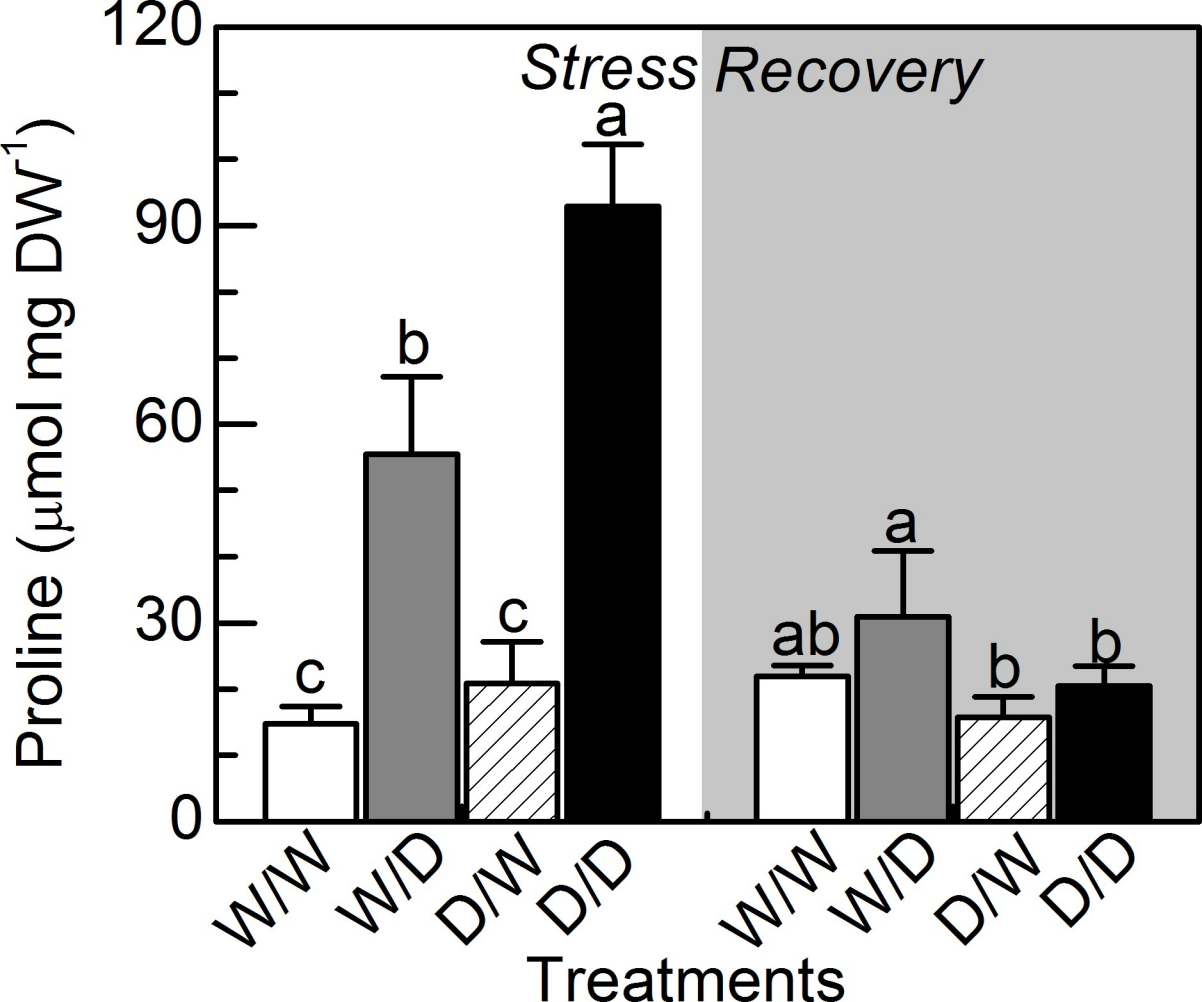
Leaf proline concentration in sugarcane propagules under water deficit. Leaf proline concentration in sugarcane plants grown under well-watered conditions (W/W and D/W) or subjected to water deficit (W/D and D/D). The propagules were obtained from plants previously exposed to water deficit (D/W and D/D) or grown under well-watered conditions (W/W and W/D). Measurements were done during the water deficit (stress) and recovery (gray area) periods, as shown in Fig 1. Each histogram is the mean value + s.d. (n = 4). Different letters mean statistical differences among treatments (p<0.05).

The leaf proline content was increased under water deficit and D/D plants presented the highest values. After the recovery period, W/D plants presented higher proline content than D/D plants (Fig 5).

**Fig 6:**
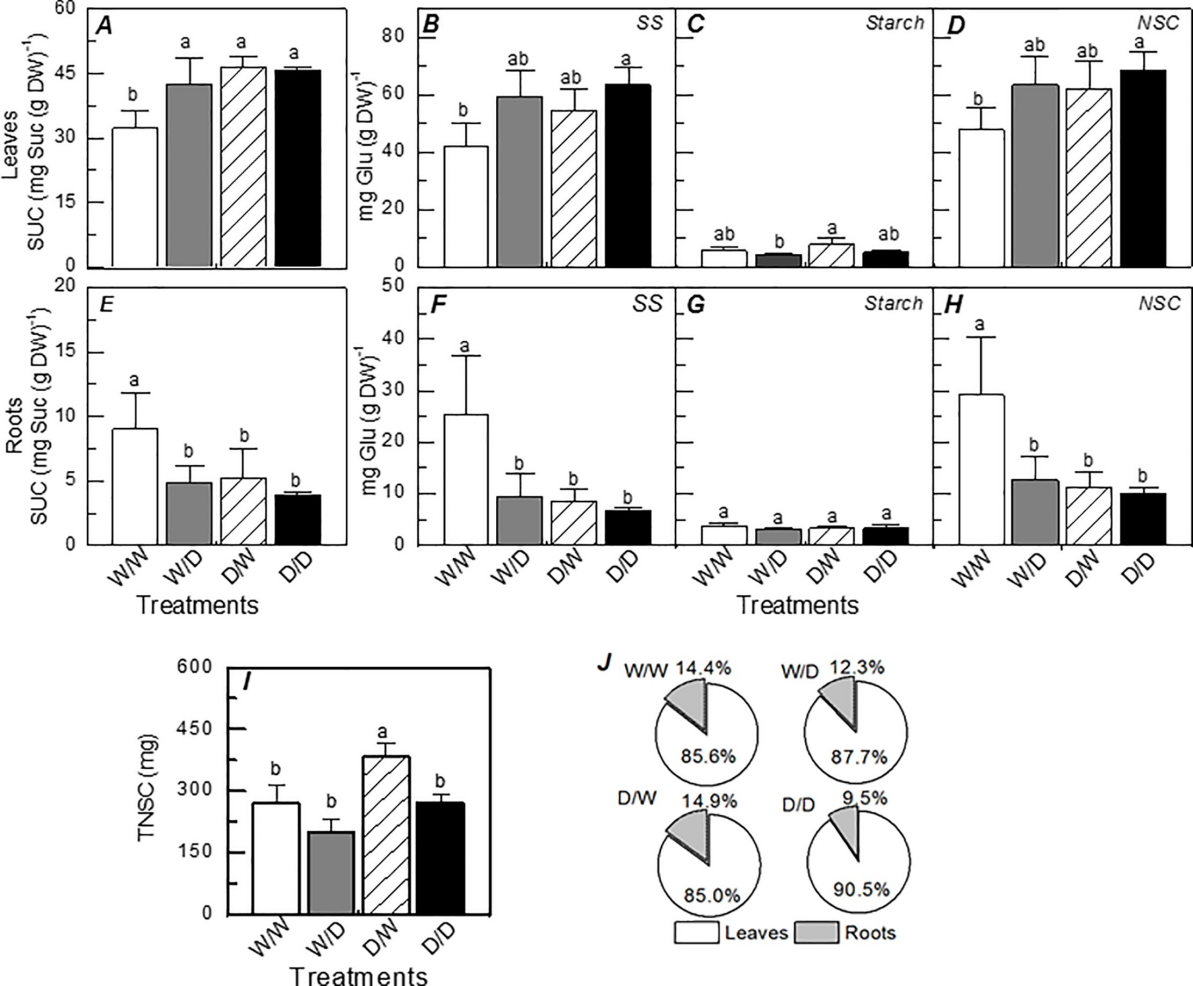
Leaf and root carbohydrates and their partitioning in sugarcane propagules under water deficit. Sucrose (*A*, *E*) soluble sugars (*B*, *F*), starch (*C*, *G*) and non-structural carbohydrates (*D*, *H*) in leaves (*A*-*D*) and roots (*E*-*H*), amount of total non-structural carbohydrates in the entire plant (*I*) and their partitioning among plant organs (*J*) in sugarcane plants grown under well-watered conditions (W/W and D/W) or subjected to water deficit (W/D and D/D). The propagules were obtained from plants previously exposed to water deficit (D/W and D/D) or grown under well-watered conditions (W/W and W/D). Measurements were taken after 9 days of water deficit (maximum water deficit). Each histogram is the mean value + s.d. (n = 4). Different letters mean statistical differences among treatments (p<0.05).

The leaf sucrose content was also increased by water deficit but only in the propagules originated from plants maintained under well-watered conditions, i.e. W/W *vs*. W/D (Fig 6A). Curiously, D/W plants had higher leaf sucrose content than W/W ones, suggesting an influence of the origin material. Such influence was also found in roots, with D/W plants presenting lower sucrose, soluble total sugars and total non-structural carbohydrates than W/W plants (Fig 6). Reductions in the root concentrations of sucrose, soluble total sugars and total non-structural carbohydrates due to water deficit were found only in the propagules obtained from plants that did not face drought (Fig 6E–6H). When considering the total amount of non-structural carbohydrates in plants (Fig 6I), D/W plants had higher values than W/W plants and the carbohydrate partitioning between leaves (86% to 91%) and roots (9% to 15%) was similar among treatments (Fig 6J).

**Fig 7:**
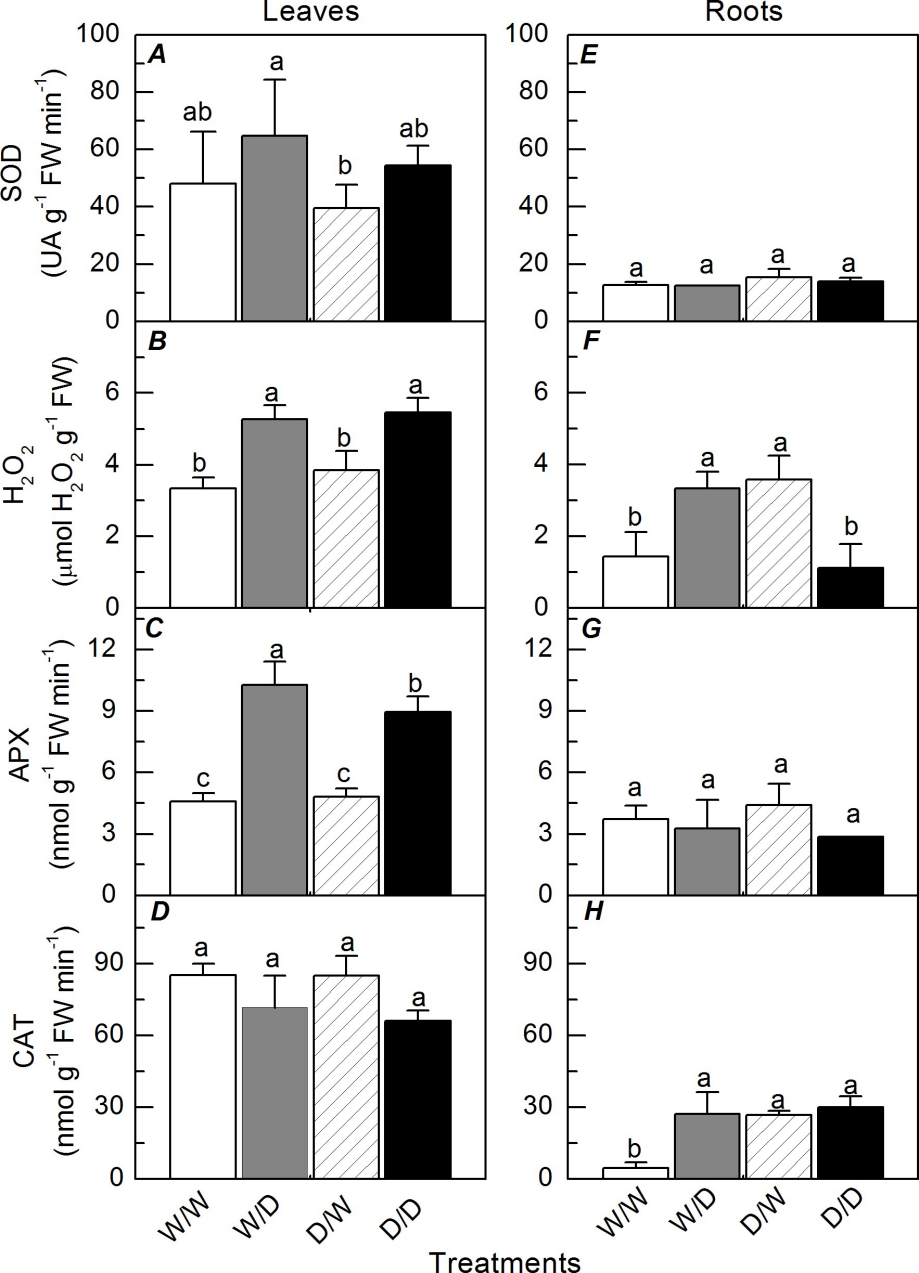
Antioxidant metabolism in sugarcane propagules under water deficit. Activities of SOD (*A*, *E*), APX (*C*, *G*), CAT (*D*, *H*) and H_2_O_2_ concentration (*B*, *F*) in leaves (*A*-*D*) and roots (*E*-*H*) of sugarcane plants grown under well-watered conditions (W/W and D/W) or subjected to water deficit (W/D and D/D). The propagules were obtained from plants previously exposed to water deficit (D/W and D/D) or grown under well-watered conditions (W/W and W/D). Measurements were taken after 9 days of water deficit (maximum water deficit). Each histogram is the mean value + s.d. (n = 4). Different letters mean statistical differences among treatments (p<0.05).

Regarding the antioxidant metabolism, leaf SOD and CAT activities were not affected either by the water regimes or the plant origin (Fig 7A and 7D), while leaf H_2_O_2_ concentration and leaf APX activity increased due to water deficit (Fig 7B and 7C). The highest leaf APX activity was found in W/D plants (Fig 7C). In roots, non-significant changes were found for SOD and APX activities (Fig 7E and 7G). The root H_2_O_2_ concentration and CAT activity increased due to water deficit in the propagules originated from well-watered plants (Fig 7F and 7H). On the other hand, the root H_2_O_2_ concentration was reduced and root CAT activity did not change under water deficit when considering the propagules originated from plants grown under cycles of water deficit (Fig 7F and 7H). Interestingly, D/W plants had higher root H_2_O_2_ concentration and higher root CAT activity than W/W plants (Fig 7F and 7H).

**Fig 8:**
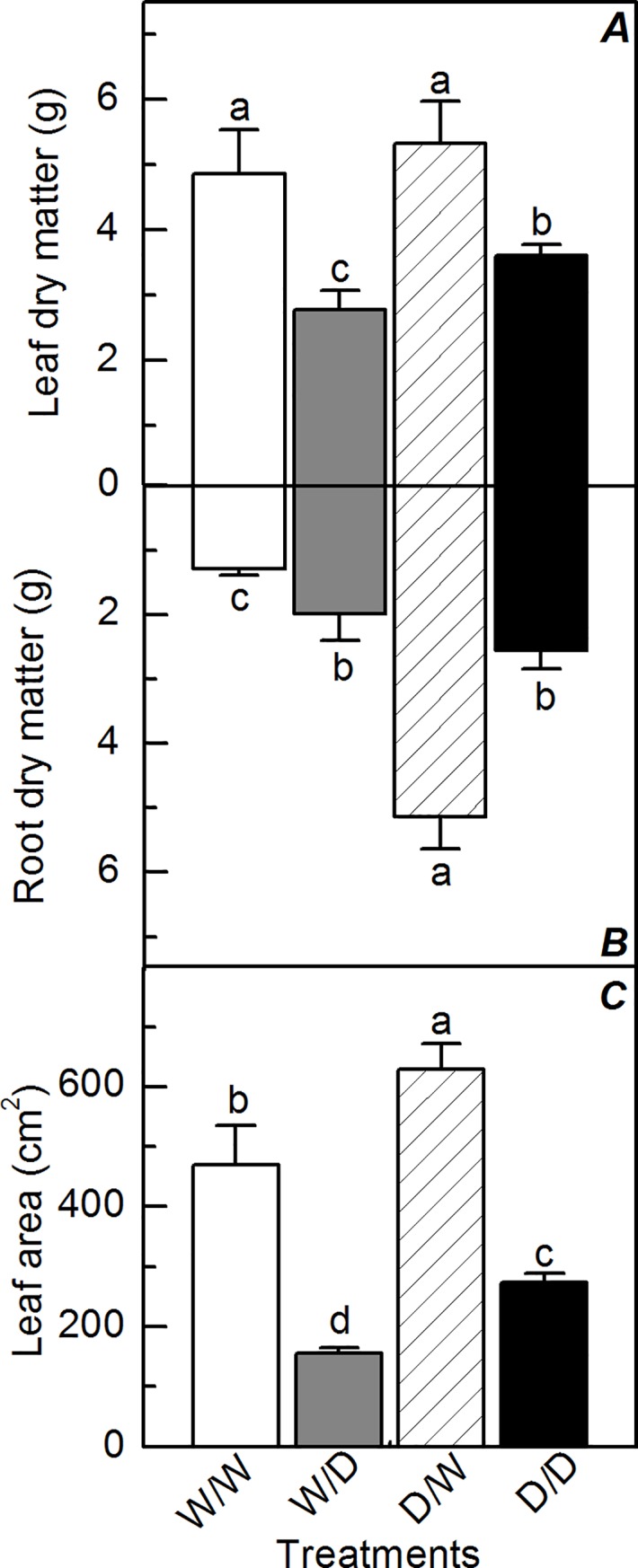
Biomass accumulation by sugarcane propagules under water deficit. Leaf (*A*) and root (*B*) dry matter and leaf area (*C*) in sugarcane plants grown under well-watered conditions (W/W and D/W) or subjected to water deficit (W/D and D/D). The propagules were obtained from plants previously exposed to water deficit (D/W and D/D) or grown under well-watered conditions (W/W and W/D). Measurements were taken at the end of experiment. Each histogram is the mean value ± s.d. (n = 4). Different letters mean statistical differences among treatments (p<0.05).

The water deficit reduced shoot biomass production regardless the plant origin, but D/D plants had higher shoot biomass than W/D plants (Fig 8A). While the propagules obtained from well-watered plants presented increases in root biomass under water deficit, the opposite was found in the propagules obtained from plants that experienced cycles of water deficit (Fig 8B). In general, root biomass of D/W plants was about four times higher than one of W/W plants, with D/D plants showing similar root biomass as compared to W/D plants. Leaf area was also reduced by the water deficit (Fig 8C), but D/D plants had higher leaf area than the propagules obtained from well-watered plants, despite the water regime.

## Discussion

Herein, we induced cycles of dehydration and rehydration to sugarcane plants, obtained propagules and tested their response to water deficit. Our initial hypothesis that plant acclimation to low water availability found in the origin material would improve the performance of propagules under unfavorable conditions was confirmed and benefits were found both in physiological reactions and in biomass production (Figs 1, 2 and 8). Interestingly, the propagules obtained from plants that faced water deficit produced more biomass than the ones from plants maintained as well-watered, regardless of the water regime (Fig 8). This suggest that the propagules obtained from stressed plants have increased their efficiency in using natural resources such as water and sunlight through the plant acclimation induced by previous exposure to water deficit.

Both plant acclimation and signaling are involved in plant memory and we have shown recently that stress memory may be induced in sugarcane plants after three cycles of water deficit, with plants showing higher photosynthesis and improved growth under water limiting conditions [5]. Those previous results together with ones reported herein indicate that the drought tolerance of sugarcane could be improved by water management and by selecting the propagation material for planting new crop fields. The propagules obtained from plants growing in areas with low water availability would be more tolerant to drought as compared to the propagules of the same genotype grown under irrigation or in areas without occurrence of water deficit.

When exposing the origin plants to water deficit, plant acclimation occurred and such information was likely stored in bud meristems, as suggested by the improved performance of plants obtained by vegetative propagation. Besides causing decreases in photosynthesis (S1 Fig) and biomass production (S2 Fig; S1 Table), cycles of dehydration and rehydration can generate a number of chemical signals, such as increases in concentration of abscisic acid (ABA), a hormone that alter the expression pattern of many genes linked to drought response [32]. Changes in gene expression patterns might be stored through DNA methylation and acetylation and induce stress memory [33], a subject poorly explored and understood in poliploid species like sugarcane. In spite of a large decrease in biomass production of the origin plants under water deficit (S2 Fig; S1 Table), their propagules had faster sprouting and higher biomass than the ones obtained from well-hydrated plants (Fig 8). Such improved plant growth was reported previously in other species, being associated with epigenetic changes [9], a research topic that should be further investigated for revealing the molecular bases of improved drought tolerance in sugarcane.

As our plants were produced from small stem segments containing one bud, we may argue that the ability of clones in recovering the stored environmental information [16] could explain both morphological and physiological responses of D/D plants. D/D plants exhibited higher photosynthesis than W/D plants at the recovery period (higher resilience) and this was caused by higher instantaneous carboxylation efficiency (Fig 1A and 1C). Regarding the primary photochemistry, the non-photochemical quenching was lower in D/D plants than in W/D plants, indicating less dissipation of energy as heat in the former ones (Fig 4D) and then that D/D plants were facing less excess of energy or energetic pressure at PSII level [32]. When considering the plant acclimation to low water availability, increases in the water use efficiency of propagules indicate an optimization of CO_2_ assimilation per unit of H_2_O transpired in D/D plants (Fig 2C). This is an important physiological change as sugarcane plants can use less soil water and then maintain soil moisture for a longer period, supporting both photoassimilate production and plant growth.

D/D plants were able to maintain metabolic activity and produce more biomass than W/D plants (Fig 8), even presenting lower leaf water potential (Fig 3A). As RWC was similar in W/D and D/D plants (Fig 3), our data indicate the occurrence of more intense osmotic adjustment in D/D plants as compared to W/D plants. This can be explained by higher concentration of proline in leaves (Fig 5), an osmotic and osmoprotectant molecule [33], with D/D plants synthesizing this osmolyte for adjusting the osmotic equilibrium and cell homeostasis during water deficit. After the rehydration, there was a large degradation of proline in D/D plants, which would increase the remobilization of nitrogen to assimilatory pathways for resuming plant growth. Regarding the osmotic adjustment and osmoprotection, the role of other molecules like glycine-betaine and dehydrins cannot be discarded as they have significant impact on plant water balance and may be chemical signals associated with plant acclimation to drought [32, 34].

Plants respond to abiotic stresses by altering their metabolism and accumulating substances such as sugars, amino acids and other metabolites with important roles in stress tolerance [35]. The maintenance of high sucrose concentration even under well-watered conditions is another example of plant acclimation to water shortage, as found in D/W plants (Fig 6A). In addition, the propagules obtained from plants that faced drought did not present any change in both leaf and root sucrose concentrations under water deficit (Fig 6A and 6E). Besides a source of energy and carbon, sucrose accumulation would improve the osmoregulation, protecting proteins and maintaining photosynthesis under low water availability [32].

Low concentrations of ROS in plants previously exposed to stressful conditions could be a consequence of physiological acclimation and suggest stress memory [36]. However, our data indicate that the exposure of origin plants to water deficit caused higher root H_2_O_2_ concentration in the propagules maintained under well-watered conditions (Fig 7F). In addition to its role in plant signaling [37], ROS accumulation is also associated with modifications in DNA methylation pattern [38] and the presence of ROS in controlled amounts is important for plant growth. In fact, plants have higher H_2_O_2_ concentration in the region of root elongation [36]. Then, high root H_2_O_2_ concentration in D/W plants (Fig 7F) would explain high root biomass (Figs 7F and 8B) as H_2_O_2_ is produced by mitochondria during the synthesis of NADH and ATP for supplying aerobic plant metabolism in active growing regions [39].

Based on the results reported herein, the next step towards the improvement of drought tolerance in sugarcane would be the evaluation of field-grown plants. In such system, not only the persistence of acclimatory changes due to previous exposure to stressors and their importance for crop yield and biomass production should be evaluated but also the genotypic variation within *Saccharum* complex in relation to drought acclimation. While the classical physiological responses to water deficit were reported herein, our data also revealed a systemic improvement in plant reaction to low water availability, with both shoots and roots of the propagules showing higher growth when they were obtained from plants previously exposed to drought. At this point, it is clear that the sugarcane buds are able to regenerate drought tolerant plants due to stress-induced signals stored in such dorment tissue.

## Conclusion

Our findings clearly show that sugarcane growth is improved in the propagules obtained from plants that faced water deficit. Such positive plant acclimation was related to physiological adjustments and may be involved in stress memory, a subject that deserves further investigation. Our data also revealed that the bud meristems of sugarcane are able to store information acquired from previous perturbation induced by water deficit. Regarding the signaling, accumulation of H_2_O_2_ in roots was associated with improved root growth in well-watered plants. The propagules also showed improved photosynthetic water use efficiency and faster recovery of photosynthesis after the rehydration. As consequence, the propagules obtained from stressed plants exhibited improvements in biomass production, regardless of the water conditions. Finally, our results bring a new perspective for the management of sugarcane fields as the plant performance could be improved under field conditions due to a large root system and higher resilience of photosynthesis after facing water shortage.

## Supporting information

S1 Fig Time course of leaf gas exchange in origin plants under water deficit. Leaf CO_2_ assimilation of plants maintained well-watered (W) or subjected to three cycles of water deficit (D). The grey area represents water withholding (nine days) and the dotted line indicates null photosynthesis. Each symbol represents the mean values ± s.d. (n = 4).
(DOCX)

S2 Fig General view of origin plants after water deficit. Visual aspect of plants grown under cycles of water deficit (left) or under well-watered conditions (right).
(DOCX)

S1 Table Biomass accumulation by origin plants under water deficit. Biometry of plants grown under well-watered (reference) conditions or subjected to cycles of water deficit. Measurements were taken after 80 days of treatment. Different letters mean statistical differences between treatments (p<0.05).
(DOCX)

## References

[1] Ribeiro RV, Machado RS, Machado EC, Machado DFSP, Magalhães filho JR, Landell MGA. Revealing drought-resistance and productive patterns in sugarcane genotypes by evaluating both physiological responses and stalk yield. Experimental Agriculture. 2013; 49, 212–224. 10.1017/S0014479712001263

[2] Sales CRG, Marchiori PER, Machado RS, Fontenele AV, Machado EC, Silveira JAG, et al Photosynthetic and antioxidant responses to drought during the sugarcane ripening? Photosynthetica. 2015; 53, 547–554. 10.1007/s11099-015-0146-x

[3] Bruce TJA, Matthes MC, Napier JA, Pickett JA. Stressful “memories” of plants: Evidence and possible mechanisms. Plant Science. 2007; 173, 603–608. 10.1016/j.plantsci.2007.09.002

[4] Galle A, Florez-Sarasa I, El Aououad H, Flexas J. (2011). The Mediterranean evergreen *Quercus ilex* and the semideciduous *Cistus albidus* differ in their leaf gas exchange regulation and acclimation to repeated drought and re-watering cycles. Journal of Experimental Botany. 2011; 62, 5207–5216. 10.1093/jxb/err233 21813795 PMC3193022

[5] Marcos FCC, Silveira NM, Mokochinski J B, Sawaya ACHF, Marchiori PER, Machado EC, et al Drought tolerance of sugarcane is improved by previous exposure to water deficit. Journal of Plant Physiology. 2018; 223, 9–18. 10.1016/j.jplph.2018.02.001 29433084

[6] Walter J, Jentsch A, Beierkuhnlein C, and Kreyling J. Ecological stress memory and cross tolerance in plants in the face of climate extremes. Environmental and Experimental Botany.2013; 94, 3–8. 10.1016/j.envexpbot.2012.02.009

[7] Izanloo A, Condon AG, Langridge P, Tester M, Schnurbusch T. Different mechanisms of adaptation to cyclic water stress in two South Australian bread wheat cultivars. Journal of Experimental Botany. 2008; 59, 3327–3346. 10.1093/jxb/ern199 18703496 PMC2529232

[8] Bresta P, Nikolopoulos D, Stavroulaki V, Vahamidis P, Economou G, Karabourniotis G. How does long-term drought acclimation modify structure-function relationships? A quantitative approach to leaf phenotypic plasticity of barley. Functional Plant Biology. 2018; 45, 1181–1194. 10.1071/FP17283 32291009

[9] Hauser MT, Aufsatz W, Jonak C, Luschnig C. Transgenerational epigenetic inheritance in plants. Biochimica et Biophysica Acta. 2011; 1809, 459–468. 10.1016/j.bbagrm.2011.03.007 21515434 PMC4359895

[10] Ding Y, Fromm M, Avramova Z. Multiple exposures to drought “train” transcriptional responses in *Arabidopsis*. Nature Communication. 2012; 3, 740 10.1038/ncomms1732 22415831

[11] Sales CRG, Ribeiro RV, Silveira JAG, Machado EC, Martins MO, Lagôa AMMA. Superoxide dismutase and ascorbate peroxidase improve the recovery of photosynthesis in sugarcane plants subjected to water deficit and low substrate temperature. Plant Physiology and Biochemistry. 2013; 73, 326–336. 10.1016/j.plaphy.2013.10.012 24184453

[12] Thellier M, Lüttge U. Plant memory: a tentative model. Plant Biology. 2012; 15, 1–12. 10.1111/j.1438-8677.2012.00674.x 23121044

[13] Chinnusamy V, Zhu JK. Epigenetic regulation of stress responses in plants. Current Opinion in Plant Biology. 2009; 12, 133–139. 10.1016/j.pbi.2008.12.006 19179104 PMC3139470

[14] Boyko A, Kovalchuk I. Genome instability and epigenetic modification—heritable responses to environmental stress? Current Opinion in Plant Biology. 2011; 14, 260–266. 10.1016/j.pbi.2011.03.003 21440490

[15] Dodd R, Douhovnikoff V. Adjusting to Global Change through Clonal Growth and Epigenetic Variation. Frontiers in Ecology and Evolution. 2016; 4, 86 10.3389/fevo.2016.00086

[16] Latzel V, Rendina González AP, Rosenthal J. Epigenetic Memory as a Basis for Intelligent Behavior in Clonal Plants. Frontiers in Plant Science. 2016; 7, 1354 10.3389/fpls.2016.01354 27630664 PMC5006084

[17] Dos Santos HG. Sistema Brasileiro de Classificação de solos. 3th ed. Brasília, DF: Embrapa; 2013.

[18] Dias FL, Rosseto R. Calagem e adubação da cana-de-açúcar. In: Atualização em produção de cana-de-açúcar, ed Segato SV, Pinto, Jendiroba A S, Nóbrega JCM. Piracicaba: Livro Ceres, 2016 pp.107–119.

[19] Sarruge JR. Soluções nutritivas. Summa Phytopathologica. 1975; 1, 231–233. http://www.scielo.br/scielo.php?script=sci_nlinks&ref=000052&pid=S0071-1276197800010001700009&lng=es

[20] Edwards GE, Baker NR. Can CO_2_ assimilation in maize leaves be predicted accurately from chlorophyll fluorescence analysis? Photosynthesis Research. 1993; 37, 89–102. 10.1007/BF02187468 24317706

[21] Baker NR. Chlorophyll Fluorescence: A probe of photosynthesis: *in vivo*. Annual Review of Plant Biology. 2008; 59, 89–113. 10.1146/annurev.arplant.59.032607.092759 18444897

[22] Weatherley PE. Studies in the water relations of the cotton plant. I. The field measurement of water deficits in leaves. New Phytologist. 1950; 49, 81–87. 10.1111/j.1469-8137.1950.tb05146.x

[23] Bieleski RL, Turner A. Separation and estimation of amino acids in crude plant extracts by thinx'-layer electrophoresis and chromatography. Analytical Biochemistry. 1966; 17, 278–293. 10.1016/0003-2697(66)90206-5 5971422

[24] Dubois M, Gilles KA, Hamilton JK, Rebers PA, Smith F. Colorimetric method for determination of sugars and related substances. Analytical Biochemistry. 1956; 28, 350–356. 10.1021/ac60111a017

[25] Van Handel E. Direct microdetermination of sucrose. Analytical Biochemistry. 1968; 22, 280–283. 10.1016/0003-2697(68)90317-5 5641848

[26] Amaral LIV, Gaspar M, Costa PMF, Aidar MPM, Buckeridge MS. Novo método enzimático rápido e sensível de extração e dosagem de amido em materiais vegetais. Hoehnea. 2007; 34, 425–431. 10.1590/S2236-89062007000400001

[27] Rena AB, Masciotti GZ. The effect of dehydration on nitrogen metabolism and growth of bean cultivars (*Phaseolus vulgaris* L.). Ceres. 1976; 23, 288–301. http://www.scielo.br/scielo.php?script=sci_nlinks&ref=000041&pid=S0006-8705198100010000500010&lng=en

[28] Alexieva V, Sergiev I, Mapelli S, and Karanov E. The effect of drought and ultraviolet radiation on growth and stress markers in pea and wheat. Plant, Cell and Environment 2001; 24, 1337–1344. 10.1046/j.1365-3040.2001.00778.x

[29] Giannopolitis O, Ries SK. Superoxide dismutase: I. Occurrence in higher plants. Plant Physiology. 1977; 59, 309–14. 10.1104/pp.59.2.309 16659839 PMC542387

[30] Havir EA, McHale NA. Biochemical and development characterization of multiples forms of catalase in Tobacco-leaves. Plant Physiology. 1987; 84, 450–455. 10.1104/pp.84.2.450 16665461 PMC1056601

[31] Nakano Y, Asada K. Hydrogen peroxide is scavenged by ascorbate-specific peroxidase in spinach chloroplasts. Plant and Cell Physiology. 1981; 22, 867–880. 10.1093/oxfordjournals.pcp.a076232

[32] Yordanov I, Velikova V, Tsonev T. Plant responses to drought, acclimation and stress tolerance. Photosynthetica. 2000; 38, 171–186. 10.1023/A:1007201411474

[33] Szabados L, Savouré A. Proline: a multifunctional amino acid. Trends in Plant Science. 2010; 15, 89–97. 10.1016/j.tplants.2009.11.009 20036181

[34] Brestic M, Zivcak M. PSII fluorescence techniques for measurement of drought and high temperature stress signal in crop plants: protocols and applications In: Molecular stress physiology of plants, ed Rout GR, Das AB. Dordrecht, Heidelberg, New York, London: Springer, 2013 pp.87–131.

[35] Verlues PE, Agarwal M, Katiyar-Agarwal S, Zhu J, Zhu J K. Methods and concepts in quantifying resistance to drought, salt and freezing, abiotic stress that affect plant water status. Plant Journal. 2006; 45, 523–539. 10.1111/j.1365-313X.2005.02593.x 16441347

[36] Hu T, Jinb Y, Lia H, Amomboa E, Fua J. Stress memory induced transcriptional and metabolic changes of perennial ryegrass (*Lolium perenne*) in response to salt stress. Physiologia Plantarum. 2015; 156, 54–69. 10.1111/ppl.12342 25913889

[37] Foyer CH, Noctor G. Oxidant and antioxidant signalling in plants: a re-evaluation of the concept of oxidative stress in a physiological context. Plant, Cell and Environment. 2005; 28, 1056–1071. 10.1111/j.1365-3040.2005.01327.x

[38] Peng H, Zang J. Plant genomic DNA methylation in response to stresses: Potential applications and challenges in plant breeding. Progress in Natural Science. 2009; 19, 1037–1045. 10.1016/j.pnsc.2008.10.014

[39] Gill SS, Tuteja N. Reactive oxygen species and antioxidant machinery in abiotic stress tolerance in crop plants. Plant Physiology and Biochemistry. 2010; 48, 909–930. 10.1016/j.plaphy.2010.08.016 20870416

