# Supplementary material for: Drought tolerance of sugarcane propagules is improved when origin material faces water deficit
Source: PLoS One. 2018 Dec 26;13(12):e0206716. doi: 10.1371/journal.pone.0206716 (PMC6306257; doi:10.1371/journal.pone.0206716)
Supplement: S1 Fig — Leaf CO2 assimilation of plants maintained well-watered (W) or subjected to three cycles of water deficit (D). The grey area represents water withholding (nine days) and the dotted line indicates null photosynthesis. Each symbol represents the mean values ± s.d. (n = 4). (DOCX) [file pone.0206716.s001.docx]

**S1 Fig.** **Time course of leaf gas exchange in origin plants under water deficit.**

Leaf CO_2_ assimilation of plants maintained well-watered (W) or subjected to three cycles of water deficit (D). The grey area represents water withholding (nine days) and the dotted line indicates null photosynthesis. Each symbol represents the mean value ± s.d. (n = 4).
