# Supplementary material for: Drought tolerance of sugarcane propagules is improved when origin material faces water deficit
Source: PLoS One. 2018 Dec 26;13(12):e0206716. doi: 10.1371/journal.pone.0206716 (PMC6306257; doi:10.1371/journal.pone.0206716)
Supplement: S2 Fig — Visual aspect of plants grown under cycles of water deficit (left) or under well-watered conditions (right). (DOCX) [file pone.0206716.s002.docx]

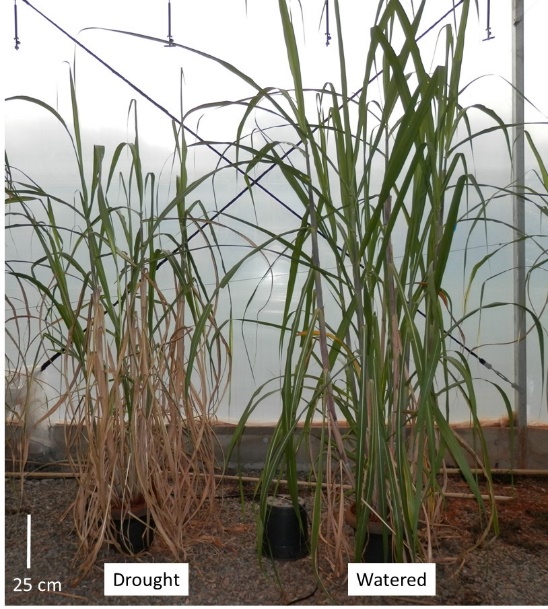


**S2 Fig**. **General view of origin plants after water deficit.**

Visual aspect of plants grown under cycles of water deficit (left) or under well-watered conditions (right).
