# Supplementary material for: Drought tolerance of sugarcane propagules is improved when origin material faces water deficit
Source: PLoS One. 2018 Dec 26;13(12):e0206716. doi: 10.1371/journal.pone.0206716 (PMC6306257; doi:10.1371/journal.pone.0206716)
Supplement: S1 Table — Biometry of plants grown under well-watered (reference) conditions or subjected to cycles of water deficit. Measurements were taken after 80 days of treatment. Different letters mean statistical differences between treatments (p<0.05). (DOCX) [file pone.0206716.s003.docx]

**S1 Table. Biomass accumulation by origin plants under water deficit.**

Biometry of plants grown under well-watered (reference) conditions or subjected to cycles of water deficit. Measurements were taken after 80 days of treatment. Different letters mean statistical differences between treatments (p<0.05).

| Variables | | Treatments | |
| --- | --- | --- | --- |
|  | Reference | | Water deficit |
| Number of green leaves (units) | 118 ± 13 a | | 38 ± 10 b |
| Number of dry leaves (units) | 44 ± 9 a | | 131 ± 20 b |
| Leaf area (m^2^) | 4.7 ± 0.3 a | | 1.2 ± 0.4 b |
| Leaf dry matter (g) | 405 ± 42 a | | 64 ± 20 b |
| Root dry matter (g) | 759 ± 263 a | | 353 ± 33 b |
| Stem dry matter (g) | 1370 ± 116 a | | 500 ± 50 b |
